# MiRNA-133b promotes the proliferation of human Sertoli cells through targeting GLI3

**DOI:** 10.18632/oncotarget.6876

**Published:** 2016-01-10

**Authors:** Chencheng Yao, Min Sun, Qingqing Yuan, Minghui Niu, Zheng Chen, Jingmei Hou, Hong Wang, Liping Wen, Yun Liu, Zheng Li, Zuping He

**Affiliations:** ^1^ State Key Laboratory of Oncogenes and Related Genes, Renji-Med X Clinical Stem Cell Research Center, Ren Ji Hospital, School of Medicine, Shanghai Jiao Tong University, Shanghai, China; ^2^ Department of Urology, Ren Ji Hospital, School of Medicine, Shanghai Jiao Tong University, Shanghai Institute of Andrology, Shanghai, China; ^3^ Shanghai Key Laboratory of Assisted Reproduction and Reproductive Genetics, Shanghai, China; ^4^ Shanghai Key Laboratory of Reproductive Medicine, Shanghai, China

**Keywords:** human Sertoli cells, global miRNA profile, Sertoli-cell-only syndrome, miRNA-133b, cell proliferation, Pathology Section

## Abstract

Sertoli cells play critical roles in regulating spermatogenesis and they can be reprogrammed to the cells of other lineages, highlighting that they have significant applications in reproductive and regenerative medicine. The fate determinations of Sertoli cells are regulated precisely by epigenetic factors. However, the expression, roles, and targets of microRNA (miRNA) in human Sertoli cells remain unknown. Here we have for the first time revealed that 174 miRNAs were distinctly expressed in human Sertoli cells between Sertoli-cell-only syndrome (SCOS) patients and obstructive azoospermia (OA) patients with normal spermatogenesis using miRNA microarrays and real time PCR, suggesting that these miRNAs may be associated with the pathogenesis of SCOS. MiR-133b is upregulated in Sertoli cells of SCOS patients compared to OA patients. Proliferation assays with miRNA mimics and inhibitors showed that miR-133b enhanced the proliferation of human Sertoli cells. Moreover, we demonstrated that GLI3 was a direct target of miR-133b and the expression of Cyclin B1 and Cyclin D1 was enhanced by miR-133b mimics but decreased by its inhibitors. Gene silencing of GLI3 using RNA inference stimulated the growth of human Sertoli cells. Collectively, miR-133b promoted the proliferation of human Sertoli cells by targeting GLI3. This study thus sheds novel insights into epigenetic regulation of human Sertoli cells and the etiology of azoospermia and offers new targets for treating male infertility

## INTRODUCTION

Non-obstructive azoospermia (NOA), which is defined as the absence of sperm in the ejaculation resulted from the testicular failure, affects about 10% of infertile men, and the incidence of NOA has been found in almost 60% of the azoospermia patients [1]. In contrast to obstructive azoospermia (OA) with normal spermatogenesis, NOA patients have aberrant spermatogenesis, e.g., maturation arrest in every stage of spermatogenesis, or a complete loss of male germ cells, which is called Sertoli-cell-only syndrome (SCOS). NOA can be caused by epigenetic and/or genetic factors, including Y chromosome microdeletions, chromosomal abnormalities, radiation, cryptorchidism, testicular torsion, varicocele, and improper drug administration [2-4]. However, the etiology causing NOA, especially SCOS, remains largely unclear.

Spermatogenesis is an intricate process, including the self-renew and differentiation of spermatogonial stem cells (SSCs), the meiosis of spermatocytes, and the spermiogenesis of haploid spermatids. These three phases are under the intimate modulation of testicular microenvironment or the ‘niche’. Sertoli cell, the only somatic cell type within the seminiferous tubules, is the most critical component of the niche [5], and it plays vital roles in regulating immunity of the seminiferous tubules and spermatogenesis. Initially, blood-testis barriers (BTB) formed by the tight junctions, adherent junctions, and gap junctions between Sertoli cells are responsible for retaining the immune privilege of the seminiferous tubules [6, 7]. Moreover, Sertoli cells secrete numerous growth factors that regulate germ cell development *via* the paracrine pathways [5, 8]. As examples, Glial cell line-derived neurotrophic factor (GDNF), produced by Sertoli cells, mediates the self-renew of SSCs [8, 9], whereas bone morphogenic protein 4 (BMP4) controls the proliferation and differentiation of SSCs [10]. In addition, stem cell factor (SCF) stimulates the proliferation of the differentiating spermatogonia and it is essential for male fertility *via* the activation of the PI3K pathway [11, 12]. We have recently revealed that SCF, BMP4, and GDNF are differentially expressed in human Sertoli cells between NOA patients and OA patients with normal spermatogenesis [13] and that BMP4 promotes the proliferation of human Sertoli cells through the Smad1/5 and ID2/3 pathway [14], which provides novel insights into genetic etiology of NOA azoospermia. Nevertheless, epigenetic regulators of NOA azoospermia remain unknown. It is worth noting that Sertoli cells could provide nutritional support for male germ cells because they can secret transferrin [15, 16] and metabolize glucose [17]. Notably, Sertoli cells have great plasticity, since they can be reprogrammed to become neural stem cells [18] and Leydig cells [19]. These studies illustrate that Sertoli cells can have important applications in regenerative medicine for treating various diseases (e.g., neural system disorders and testosterone deficiency during the aging of men). However, the epigenetic regulation of human Sertoli cells needs to be clarified.

MicroRNA (miRNA), a new class of endogenous small RNA molecules (18-22 nucleotides in length) can negatively regulate gene expression either by targeting mRNA for degradation or by translation inhibition. It has been elucidated that miRNAs play critical roles in the development of male germ cells [20]. We have recently uncovered that 559 miRNAs are distinctively expressed among human spermatogonia, pachytene spermatocytes, and round spermatids [21], suggesting that these miRNAs may have essential function in regulating the mitosis, meiosis, and spermiogenesis. It has been reported that Sertoli cell specific deletion of Dicer, a central component of the RNA interference machinery, severely impairs Sertoli cell competence, which leads to male infertility due to the absence of mature spermatozoa and testicular degeneration, reflecting that miRNAs in Sertoli cells are essential for normal spermatogenesis [22]. However, the expression, roles, and targets of miRNAs in human Sertoli cells remain unknown.

In this study, we have for the first time reported that 174 miRNAs were distinctly expressed in human Sertoli cells between SCOS patients and OA patients with normal spermatogenesis. We found that miR-133b was upregulated in human Sertoli cells of SCOS patients compared to OA patients. It has been reported that miR-133b plays a vital role in regulating the proliferation of the cancer cells [23] and it is involved in the oocyte growth and maturation [24]. However, the function and targets of miR-133b in regulating male reproduction are still unclear. Cellular and molecular assays demonstrated that miR-133b promoted the proliferation of human Sertoli cells *via* targeting transcription factor GLI3 (GLI family zinc finger 3) and activating Cyclin B1 and Cyclin D1. Significantly, this study could offer new epigenetic mechanisms controlling the fate determinations of human Sertoli cells, and it could provide new targets for gene therapy of male infertility and for their applications in regenerative medicine.

## RESULTS

### Isolation and identification of human Sertoli cells

Human Sertoli cells were isolated from OA patients and SCOS patients using a two-step enzymatic digestion and followed by differential plating as previously described [25]. The viability of freshly isolated cells was over 96%, as evidenced by trypan blue exclusion (data not shown). After removing male germ cells, human Sertoli cells were cultured in DMEM/F12 supplemented with 10% FBS for one day.

The freshly isolated human Sertoli cells were identified by various markers of Sertoli cells. RT-PCR showed that the transcripts of *GATA4*, *WT1*, *SOX9*, *GDNF*, *SCF*, *BMP4*, *FSHR,* and *AR* were expressed in the isolated Sertoli cells (Supplementary Figure 1A). RNA samples without RT (RT-) were performed with PCR reactions, and no production was observed (Supplementary Figure 1A), thus demonstrating specific expression of these genes in the isolated Sertoli cells. Immunocytochemistry further revealed that the freshly isolated human cells were positive for GATA4 (Supplementary Figure 1B), WT1 (Supplementary Figure 1C), SOX9 (Supplementary Figure 1D), BMP4 (Supplementary Figure 1E), SCF (Supplementary Figure 1F) and GDNF (Supplementary Figure 1G). Replacement of primary antibodies with goat IgG (Supplementary Figure 1H) or rabbit IgG (Supplementary Figure 1I), and no positive staining was seen, thus reflecting specific expression of these proteins in the isolated human Sertoli cells. In addition, very few cells were positive for CYP11A1 (Supplementary Figure 1J) or SMA (Supplementary Figure 1K), hallmarks for Leydig cells and myoid cells [26, 27], respectively. The purity of isolated human Sertoli cells was more than 98%, as assessed by the expression of these specific markers for Sertoli cells. Taken together, these results suggest that the isolated cells were human Sertoli cells in phenotype.

### Distinct global miRNA profiles in human Sertoli cells between SCOS patients and OA patients with normal spermatogenesis

**Figure 1:**
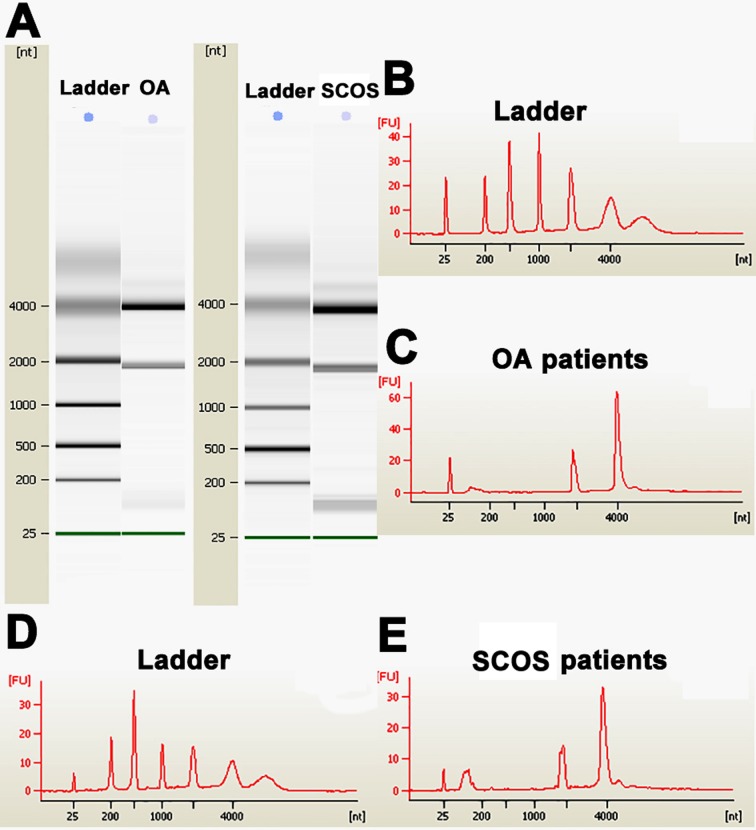
The quality evaluation of total RNA of human Sertoli cells derived from SCOS patients and OA patients **A.** Gel imaging showed the integrity of total RNA used for miRNA microarrays. **B.**-**E.** Electropherogram by Agilent bioanalyzer displayed the concentrations and nucleotides (nt) of RNA isolated from human Sertoli cells of OA patients **C.** and SCOS patients **E.**. RNA ladders **B.** and **D.** were shown as references.

**Figure 2:**
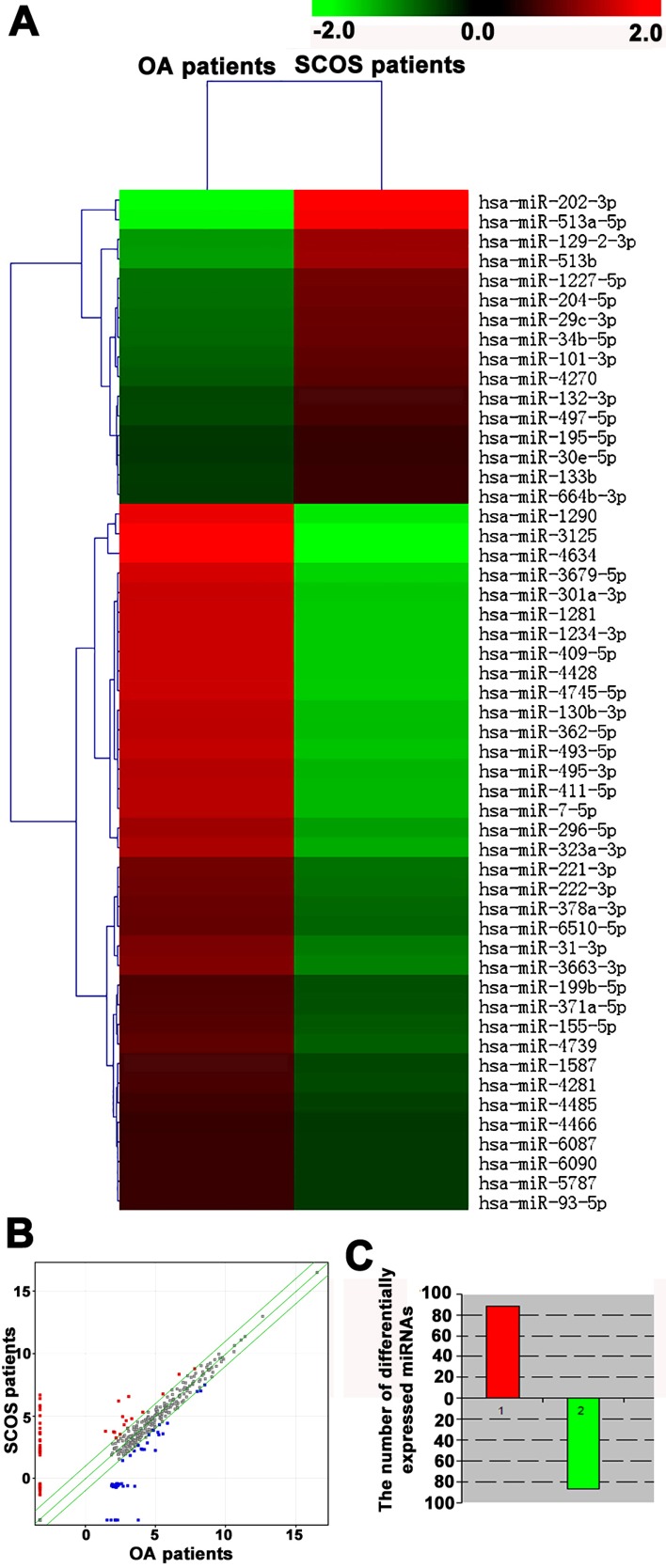
MiRNA microarrays revealed distinct global miRNA expression profiles in human Sertoli cells between OA patients and SCOS patients **A.** Hierarchical clustering analysis showed the differentially expressed miRNAs in human Sertoli cells between SCOS patients and OA patients. In total, 174 differentially expressed miRNAs were found in term of 1.5-fold and greater differences. Upregulated and downregulated miRNAs were indicated in red and green, respectively. **B.** Scatter plots revealed the patterns of miRNAs in human Sertoli cells between SCOS patients and OA patients. The log_2_ scales of the expression signal values were plotted for all probes, excluding the control and flagged probes. Standard selection criteria to identify differentially expressed miRNAs was established at log_2_ (Fold change) ≥ 0.585 and *p*-value < 0.05 (red dots and blue dots). **C.** Histogram plots displayed the number of differentially expressed miRNAs in human Sertoli cells between SCOS patients and OA patients. Red indicated upregulated miRNAs, whereas green denoted downregulated miRNAs.

After the isolation and characterization of human Sertoli cells, total RNA was extracted from human Sertoli cells of OA patients and SCOS patients. High quality of RNA for miRNA microarrays was shown by gel imaging (Figure 1A) and electrocardiogram (Figure 1B-1E). RNA samples with RIN values of more than 7.0 were used in this study. MiRNA microarrays were conducted to compare global miRNA profiles in human Sertoli cells between OA patients and SCOS patients. Hierarchical clustering analysis revealed distinct miRNA expression profiles in human Sertoli cells between OA patients and SCOS patients (Figure 2A). Based on 2,400 miRNAs in the miRNA microarray database, there were 174 differentially expressed miRNAs with 1.5-fold changes or more between these two cell populations. Scatter plots comparison revealed distinct miRNAs patterns in human Sertoli cells between SCOS patients and OA patients (Figure 2B). The log_2_ scales of expression signal values were plotted for all miRNA probes excluding the control and flagged probes in human Sertoli cells between SCOS patients and OA patients. Red dots represented the upregulated miRNAs, whereas blue dots indicated the downregulated miRNAs. In parallel, the gray dots denoted miRNAs with no significantly statistical difference. Specifically, 88 miRNAs were upregulated (Figure 2C, Table 4) whereas 86 miRNAs were downregulated in human Sertoli cells between SCOS patients and OA patients (Figure 2C, Table 5), suggesting that these miRNAs may be involved in the etiology of SCOS.

**Table 1:** The sequences of gene primers used for RT-PCR and real-time PCR

Genes	Primer sequences		Product size (bp)	Tm(°C)
***GATA4***	Forward	GCCTCCTCTGCCTGGTAAT	210	60
	Reverse	CAGTCCCATCAGCGTGTAAA		
***WT1***	Forward	TGACTCTCCACTCCTCCTCAC	170	60
	Reverse	ACCAACTCTTCCAGGCACAC		
***SOX9***	Forward	AGGTGCTCAAAGGCTACGACTG	322	60
	Reverse	TGCCCGTTCTTCACCGACT		
***GDNF***	Forward	GAAGTTATGGGATGTCGTG	419	58
	Reverse	TCAGTTCCTCCTTGGTTTC		
***SCF***	Forward	GTCATTGTTGGATAAGCGAGAT	457	60
	Reverse	ATGGCTGCCCAGTGTAGG		
***BMP4***	Forward	TTTGTTCAAGATTGGCTGTC	324	60
	Reverse	AGATCCCGCATGTAGTCC		
***FSHR***	Forward	TCTGCTGGTTCTGTTTCA	215	58
	Reverse	CATTCCTTGGATGGGTGT		
***AR***	Forward	CCTTCACCAATGTCAACTCC	197	60
	Reverse	CCACTGGAATAATGCTGAAGAG		
***ACTB***	Forward	CGCACCACTGGCATTGTCAT	253	55
	Reverse	TTCTCCTTGATGTCACGCAC		
***GLI3***	Forward	AGGGTGAATGGTATCAAGATGG	98	60
	Reverse	CCCACGGTTTGGTCATAGAA		
***GAPDH***	Forward	CAGGAGGCATTGCTGATGAT	138	60
	Reverse	GAAGGCTGGGGCTCATTT		
***ACTB***	Forward	CCTGGCACCCAGCACAAT	144	60
	Reverse	GGGCCGGACTCGTCATAC		

**Table 2:** The sequences of miRNA specific primers used for real-time PCR

Human miRNAs	Primer sequences	Tm(°C)
**hsa-miR-133b**	TTTGGTCCCCTTCAACCAGCTA	60
**hsa-miR-204-5p**	TTCCCTTTGTCATCCTATGCCT	60
**hsa-miR-30e-5p**	TGTAAACATCCTTGACTGGAAG	60
**hsa-miR-4270**	TCAGGGAGTCAGGGGAGGGC	60
**hsa-miR-129-2-3p**	AAGCCCTTACCCCAAAAAGCAT	60
**hsa-miR-202-3p**	AGAGGTATAGGGCATGGGAA	60
**hsa-miR-195-5p**	TAGCAGCACAGAAATATTGGC	60
**hsa-miR-664b-3p**	TTCATTTGCCTCCCAGCCTACA	60
**hsa-miR-497-5p**	CAGCAGCACACTGTGGTTTGT	60
**hsa-miR-34b-5p**	TAGGCAGTGTCATTAGCTGATTG	60
**hsa-miR-513a-5p**	TTCACAGGGAGGTGTCAT	60
**hsa-miR-101-3p**	TACAGTACTGTGATAACTGAA	60
**hsa-miR-221-3p**	AGCTACATTGTCTGCTGGGTTTC	60
**hsa-miR-409-5p**	AGGTTACCCGAGCAACTTTGCAT	60
hsa-miR-1290	TGGATTTTTGGATCAGGGA	60
**hsa-miR-155-5p**	TTAATGCTAATCGTGATAGGGGT	60
**hsa-miR-31-3p**	TGCTATGCCAACATATTGCCAT	60
**hsa-miR-7-5p**	TGGAAGACTAGTGATTTTGTTGT	60
**hsa-miR-362-5p**	AATCCTTGGAACCTAGGTGTGAGT	60
**hsa-miR-493-5p**	TTGTACATGGTAGGCTTTCATT	60
**hsa-miR-296-5p**	AGGGCCCCCCCTCAATCCTGT	60
**hsa-miR-199b-5p**	CCCAGTGTTTAGACTATCTGTTC	60
**U6**	CAAGGATGACACGCAAATTCG	60

**Table 3:** The sequences of oligonucleotides in miRNA mimics, miRNA inhibitor and siRNAs

Oligonucleotides	Sequences(5′-3′)	
MiRNA mimics control	Sense	UUCUCCGAACGUGUCACGUTT
	Antisense	ACGUGACACGUUCGGAGAATT
MiRNA inhibitor control		CAGUACUUUUGUGUAGUACAA
Hsa-miR-133b mimics	Sense	UUUGGUCCCCUUCAACCAGCUA
	Antisense	GCUGGUUGAAGGGGACCAAAUU
Hsa-miR-133b inhibitor		UAGCUGGUUGAAGGGGACCAAA
Control siRNA	Sense	UUCUCCGAACGUGUCACGUTT
	Antisense	ACGUGACACGUUCGGAGAATT
GLI3 siRNA-1	Sense	GUCUCGUGCUUCAGAAUUATT
	Antisense	UAAUUCUGAAGCACGAGACTT
GLI3 siRNA-2	Sense	GCCCAGCAGAAUACUAUCATT
	Antisense	UGAUAGUAUUCUGCUGGGCTT
GLI3 siRNA-3	Sense	GGUCACGAUUCUCAAUAAUTT
	Antisense	AUUAUUGAGAAUCGUGACCTT
GAPDH siRNA	Sense	UGACCUCAACUACAUGGUUTT
	Antisense	AACCAUGUAGUUGAGGUCATT

**Table 4:** Representative upregulated miRNAs in human Sertoli cells between SCOS patients and OA patients

Systematic name	SCOS Probe Signal (normalized)	OA Probe Signal (normalized)	Log^2^ FC (SCOS *vs*. OA)
hsa-miR-1	2.536125	−3.24945	5.785577
hsa-miR-101-3p	4.301949	2.767519	1.53443
hsa-miR-1185-2-3p	−0.6910555	−3.24945	2.558397
hsa-miR-1227-5p	3.748628	1.997267	1.751361
hsa-miR-1271-5p	−0.57203066	−3.24945	2.677422
hsa-miR-128	−0.55576026	−3.24945	2.693692
hsa-miR-129-2-3p	4.9401026	2.658367	2.281735
hsa-miR-129-5p	2.1114187	−3.24945	5.360871
hsa-miR-132-3p	5.289028	4.08851	1.200518
hsa-miR-133b	3.2301393	2.186946	1.043193
hsa-miR-135b-5p	2.9981375	−3.24945	6.24759
hsa-miR-136-3p	−0.6821088	−3.24945	2.567344
hsa-miR-146b-5p	2.393536	−3.24945	5.642988
hsa-miR-148b-3p	−0.7858837	−3.24945	2.463569
hsa-miR-154-3p	2.0743582	−3.24945	5.323811
hsa-miR-17-3p	−0.6714288	−3.24945	2.578023
hsa-miR-192-5p	−0.664656	−3.24945	2.584796
hsa-miR-193b-5p	−0.75815064	−3.24945	2.491302
hsa-miR-195-5p	8.798399	7.790861	1.007538
hsa-miR-202-3p	6.192669	2.362819	3.82985
hsa-miR-204-5p	4.6164274	2.893112	1.723315
hsa-miR-214-5p	−0.749807	−3.24945	2.499645
hsa-miR-22-5p	−0.67559606	−3.24945	2.573856
hsa-miR-23b-5p	−0.9447008	−3.24945	2.304752
hsa-miR-24-1-5p	2.0840352	−3.24945	5.333488
hsa-miR-29c-3p	8.335577	6.67212	1.663457
hsa-miR-29c-5p	−0.67475003	−3.24945	2.574702
hsa-miR-30a-3p	1.8863611	−3.24945	5.135814
hsa-miR-30e-3p	−0.7797832	−3.24945	2.469669
hsa-miR-30e-5p	4.0255494	3.022563	1.002986
hsa-miR-3141	−0.60127103	−3.24945	2.648181
hsa-miR-339-3p	−1.314496	−3.24945	1.934956
hsa-miR-34b-5p	3.6975255	2.074358	1.623167
hsa-miR-34c-5p	−0.55098486	−3.24945	2.698468
hsa-miR-362-3p	−0.46611047	−3.24945	2.783342
hsa-miR-376b-3p	−0.6631639	−3.24945	2.586289
hsa-miR-4252	2.2096176	−3.24945	5.45907
hsa-miR-4270	4.7751856	3.317884	1.457302
hsa-miR-4433-3p	−0.708564	−3.24945	2.540888
hsa-miR-4449	−0.42239857	−3.24945	2.827054
hsa-miR-451b	3.0789175	−3.24945	6.32837
hsa-miR-455-5p	−0.6786612	−3.24945	2.570791
hsa-miR-4633-5p	−0.7354823	−3.24945	2.51397
hsa-miR-4730	4.034162	−3.24945	7.283614
hsa-miR-487a	−0.6068021	−3.24945	2.64265
hsa-miR-497-5p	6.750507	5.512681	1.237826
hsa-miR-5010-3p	−0.6420094	−3.24945	2.607443
hsa-miR-502-3p	−0.61375463	−3.24945	2.635698
hsa-miR-506-3p	5.503647	−3.24945	8.753099
hsa-miR-507	3.5694683	−3.24945	6.818921
hsa-miR-508-3p	3.6531644	−3.24945	6.902617
hsa-miR-508-5p	−1.0321872	−3.24945	2.217265
hsa-miR-509-3-5p	5.7557783	−3.24945	9.005231
hsa-miR-509-3p	4.4269204	−3.24945	7.676373
hsa-miR-509-5p	5.852409	−3.24945	9.101861
hsa-miR-510	3.7108288	−3.24945	6.960281
hsa-miR-513a-5p	6.559836	3.087263	3.472573
hsa-miR-513b	3.7757204	1.439532	2.336188
hsa-miR-513c-5p	5.036705	−3.24945	8.286158
hsa-miR-514a-3p	6.395572	−3.24945	9.645024
hsa-miR-514a-5p	2.2883399	−3.24945	5.537792
hsa-miR-514b-5p	6.681776	−3.24945	9.931229
hsa-miR-532-3p	−0.48485875	−3.24945	2.764594
hsa-miR-539-5p	−0.6521455	−3.24945	2.597307
hsa-miR-557	2.6437259	−3.24945	5.893178
hsa-miR-6515-3p	2.3628187	−3.24945	5.612271
hsa-miR-664b-3p	3.5130694	2.481298	1.031771
hsa-miR-769-5p	−0.57381034	−3.24945	2.675642
hsa-miR-873-5p	−1.2615904	−3.24945	1.987862

**Table 5:** Representative downregulated miRNAs in human Sertoli cells between SCOS patients and OA patients

Systematic name	SCOS Probe Signal (normalized)	OA Probe Signal (normalized)	Log^2^ FC (SCOS *vs*. OA)
hsa-miR-1234-3p	−0.54959	2.345284	−2.89488
hsa-miR-1281	−0.69541	2.193778	−2.88919
hsa-miR-1290	−0.61634	2.692763	−3.3091
hsa-miR-130b-3p	2.25165	4.984564	−2.73291
hsa-miR-155-5p	4.422804	5.839585	−1.41678
hsa-miR-1587	1.439532	2.643726	−1.20419
hsa-miR-199b-5p	2.186946	3.493466	−1.30652
hsa-miR-221-3p	3.493466	5.251211	−1.75774
hsa-miR-222-3p	2.325802	4.047785	−1.72198
hsa-miR-296-5p	−0.46595	1.886361	−2.35232
hsa-miR-301a-3p	−0.65145	2.209618	−2.86107
hsa-miR-31-3p	2.315457	4.184875	−1.86942
hsa-miR-3125	−0.5903	3.01036	−3.60066
hsa-miR-323a-3p	−0.61147	1.883982	−2.49546
hsa-miR-362-5p	−0.43678	2.283441	−2.72022
hsa-miR-3663-3p	3.571862	5.543531	−1.97167
hsa-miR-3679-5p	−0.44247	2.59451	−3.03698
hsa-miR-371a-5p	1.842062	3.181407	−1.33935
hsa-miR-378a-3p	2.345284	3.981603	−1.63632
hsa-miR-409-5p	−0.47099	2.421606	−2.89259
hsa-miR-411-5p	−0.42537	2.233557	−2.65893
hsa-miR-4281	6.987801	8.236823	−1.24902
hsa-miR-4428	−0.72234	2.177488	−2.89983
hsa-miR-4466	4.282579	5.289028	−1.00645
hsa-miR-4485	2.658367	3.786567	−1.1282
hsa-miR-4634	−0.64296	3.527475	−4.17044
hsa-miR-4739	3.672723	5.179572	−1.50685
hsa-miR-4745-5p	−0.60021	2.315457	−2.91566
hsa-miR-493-5p	−0.5903	2.21381	−2.80411
hsa-miR-495-3p	−0.49876	2.136437	−2.63519
hsa-miR-5787	3.503378	4.52326	−1.01988
hsa-miR-6087	6.953431	7.980244	−1.02681
hsa-miR-6090	7.484371	8.512548	−1.02818
hsa-miR-6510-5p	2.83112	4.42692	−1.5958
hsa-miR-7-5p	−0.69092	1.970514	−2.66144
hsa-miR-93-5p	3.837303	4.857638	−1.02033

### Quantitative real-time PCR verified the differentially expressed miRNAs in human Sertoli cells between SCOS patients and OA patients identified by miRNA microarrays

**Figure 3:**
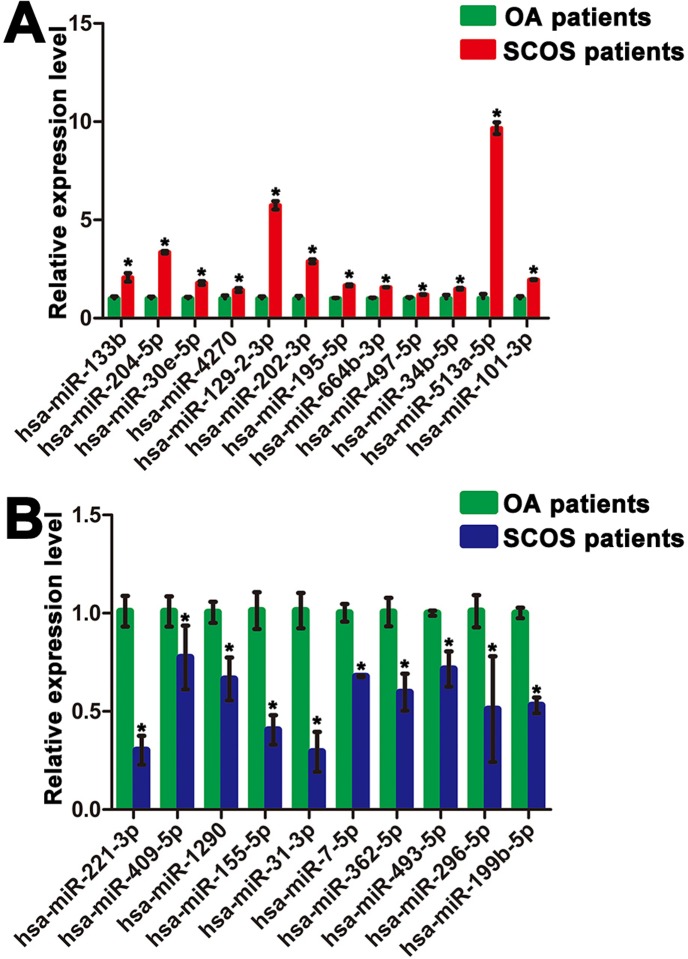
Distinct expression patterns of miRNAs in human Sertoli cells between OA patients and SCOS patients **A.** Real-time PCR showed that the expression of human miR-133b, miR-204-5p, miR-30e-5p, miR-4270, miR-129-2-3p, miR-202-3p, miR-195-5p, miR-664b-3p, miR-497-5p, miR-34b-5p, miR-513a-5p, and miR-101-3p was statistically higher in Sertoli cells of SCOS patients than Sertoli cells of OA patients. **B.** Real-time PCR further revealed that human miR-221-3p, miR-409-5p, miR-1290, miR-155-5p, miR-31-3p, miR-7-5p, miR-362-5p, miR-493-5p, miR-296-5p, and miR-199b-5p were statistically expressed at lower levels in Sertoli cells of SCOS patients than Sertoli cells of OA patients. * indicated statistically significant differences (*p* < 0.05) in human Sertoli cells between SCOS patients and OA patients.

In order to verify the data of miRNA microarrays, we further conducted the quantitative real-time PCR for a number of the differentially expressed miRNAs in human Sertoli cells between SCOS patients and OA patients. In total, 22 distinctly expressed miRNAs were chosen randomly for the real-time PCR according to their fold changes in these two cell populations. Real-time PCR revealed that hsa-miR-133b, hsa-miR-204-5p, hsa-miR-30e-5p, hsa-miR-4270, hsa-miR-129-2-3p, hsa-miR-202-3p, hsa-miR-195-5p, hsa-miR-664b-3p, hsa-miR-497-5p, hsa-miR-34b-5p, hsa-miR-513a-5p, and hsa-miR-101-3p were statistically upregulated in human Sertoli cells of SCOS patients compared to OA patients (Figure 3A). In contrast, hsa-miR-221-3p, hsa-miR-409-5p, hsa-miR-1290, hsa-miR-155-5p, hsa-miR-31-3p, hsa-miR-7-5p, hsa-miR-362-5p, hsa-miR-493-5p, hsa-miR-296-5p, and hsa-miR-199b-5p were statistically downregulated in human Sertoli cells of SCOS patients compared to OA patients (Figure 3B). The data of real-time PCR were fully consistent with the expression patterns of these miRNAs by our miRNA microarrays.

### MiR-133b promoted the proliferation of human Sertoli cells

**Figure 4:**
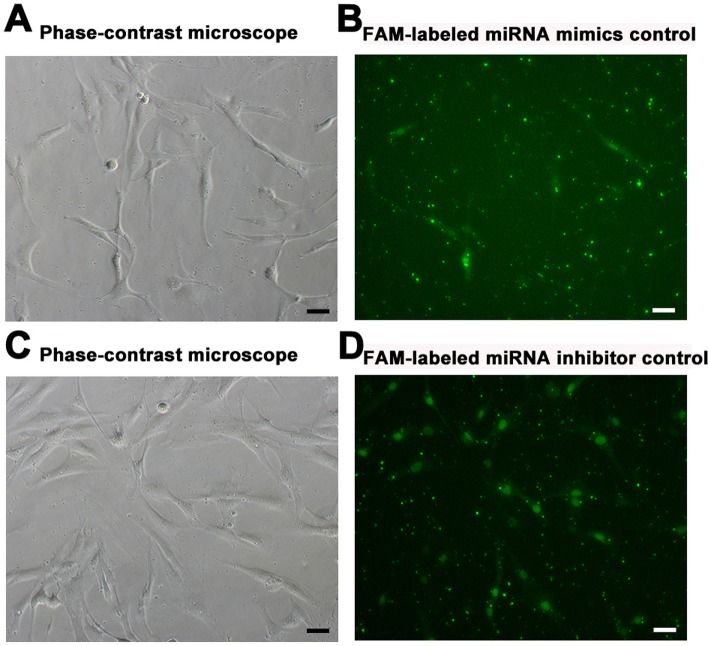
Transfection efficiency of miR-133b mimics and inhibitor in human Sertoli cells **A.**-**B.** Phase-contrast microscope. **A.** and fluorescence microscope **B.** showed the transfection efficiency of miR-133b mimics using the FAM-labeled miRNA mimics control oligonucleotides. **C.**-**D.** Phase-contrast microscope **C.** and fluorescence microscope **D.** displayed the transfection efficiency of miR-133b inhibitor using the FAM-labeled miRNA inhibitor control oligonucleotides. Scale bars in A-D = 20 μm.

Since hsa-miR-133b was statistically upregulated in human Sertoli cells of SCOS patients compared to OA patients, as shown by our miRNA microarrays and real-time PCR, we further explored the function of miR-133b in the regulation of human Sertoli cells using miR-133b mimics and inhibitors. The transfection efficiency of miRNA-133b mimics or inhibitors in human Sertoli cells was around 75%, as assessed by transfection of FAM-labeled miRNA mimic control (Figure 4A-4B) and FAM-labeled miRNA inhibitor control (Figure 4C-4D).

**Figure 5:**
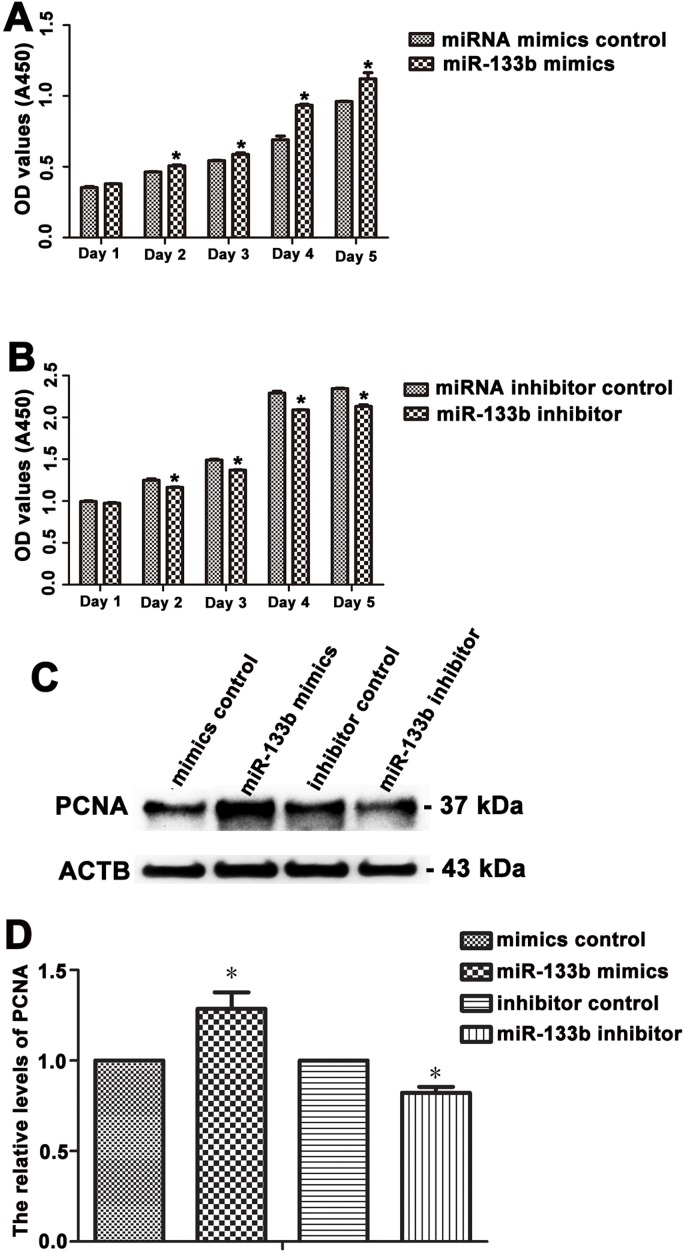
The effect of miR-133b on the proliferation of human Sertoli cells **A.**-**B.** CCK-8 assay showed the growth curve of human Sertoli cells treated with miRNA mimics control and miR-133b mimics for 5 days **A.** or miRNA inhibitor control and miR-133b inhibitor for 5 days **B.**. * indicated statistically significant differences (*p* < 0.05) between miRNA-treated group and the control. **C.** Western blots revealed that PCNA expression in human Sertoli cells at day 3 after transfection of miRNA mimics control, miR-133b mimics, miRNA inhibitor control, and miR-133b inhibitor. ACTB served as the control of loading proteins. **D.** The relative expression of PCNA in human Sertoli cells at day 3 after transfection of miR-133b mimics to miRNA mimics control and miR-133b inhibitor to miRNA inhibitor control after normalization to the signals of their loading control. * indicated statistically significant differences (*p* < 0.05) between miRNA-treated group and the control.

Cell proliferation assays were conducted at 24 hours to 120 hours in human Sertoli cells after transfection of miR-133b mimics or miR-133b inhibitor. Notably, miR-133b mimics enhanced the growth of human Sertoli cells significantly compared to miRNA mimic control in a time-dependent manner (Figure 5A). In contrast, miR-133b inhibitor statistically reduced cell number of human Sertoli cells compared with miRNA inhibitor control (Figure 5B). PCNA (Proliferating cell nuclear antigen) has been generally regarded as a hallmark for cellular proliferation [14]. Western blots revealed that the expression of PCNA was obviously increased by miR-133b mimics (1.285±0.159) compared to miRNA mimic control (designed as 1.0) in human Sertoli cells whereas its expression was significantly decreased by miR-133b inhibitor (0.822±0.055) when compared with miRNA inhibitor control (designed as 1.0) in human Sertoli cells (Figure 5C-5D). To test whether miR-133b has general effect on cell proliferation, we examined its influence on human SSC line [28]. No statistical difference was observed in cell number of human SSC line between the miR-133b mimics and miRNA mimics control (Supplementary Figurere 2A) or the miR-133b inhibitor and miRNA inhibitor control (Supplementary Figurere 2B), thus verifying a specific role of miR-133b in human Sertoli cells.

**Figure 6:**
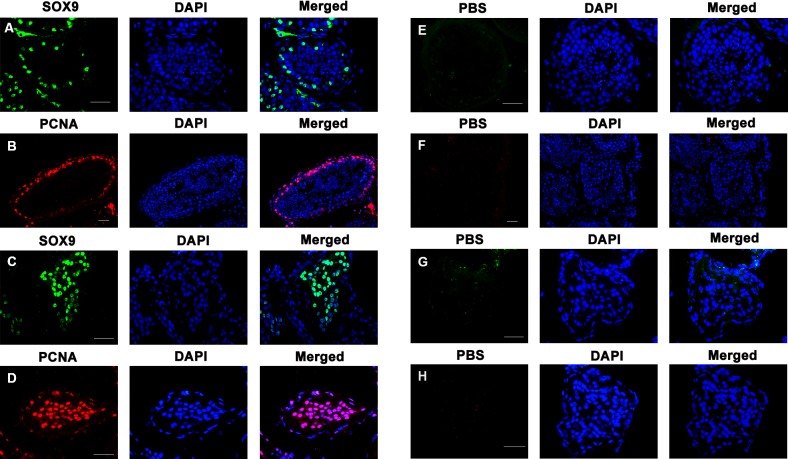
Expression of SOX9 and PCNA proteins in the testis of OA and SCOS patients **A.**-**B.** Immunohistochemical staining showed the expression of SOX9 **A.** and PCNA proteins **B.** in the testis of OA patients. **C.**-**D.** Immunohistochemistry revealed the expression of SOX9 and PCNA in the testis of SCOS patients. **E.**-**H.** Negative controls used PBS without primary antibody in the testes of OA **E.**-**F.** and SCOS patients **G.**-**H.**. Scale bars in A-H = 50 μm.

In addition, immunohistochemistry showed that more cells were positive for SOX9 and PCNA in SCOS patients (Figure 6C-6D) than OA patients with normal spermatogenesis (Figure 6A-6B), suggesting that more human Sertoli cells proliferate in SCOS patients than OA patients. Replacement of antibodies to SOX9 and PCNA with PBS, and no staining was seen in the testis of SCOS patients and OA patients (Figure 6E-6H), thus confirming specific staining of SOX9 and PCNA in the testis of these patients.

### GLI3 was a direct target of miR-133b in human Sertoli cells

**Figure 7:**
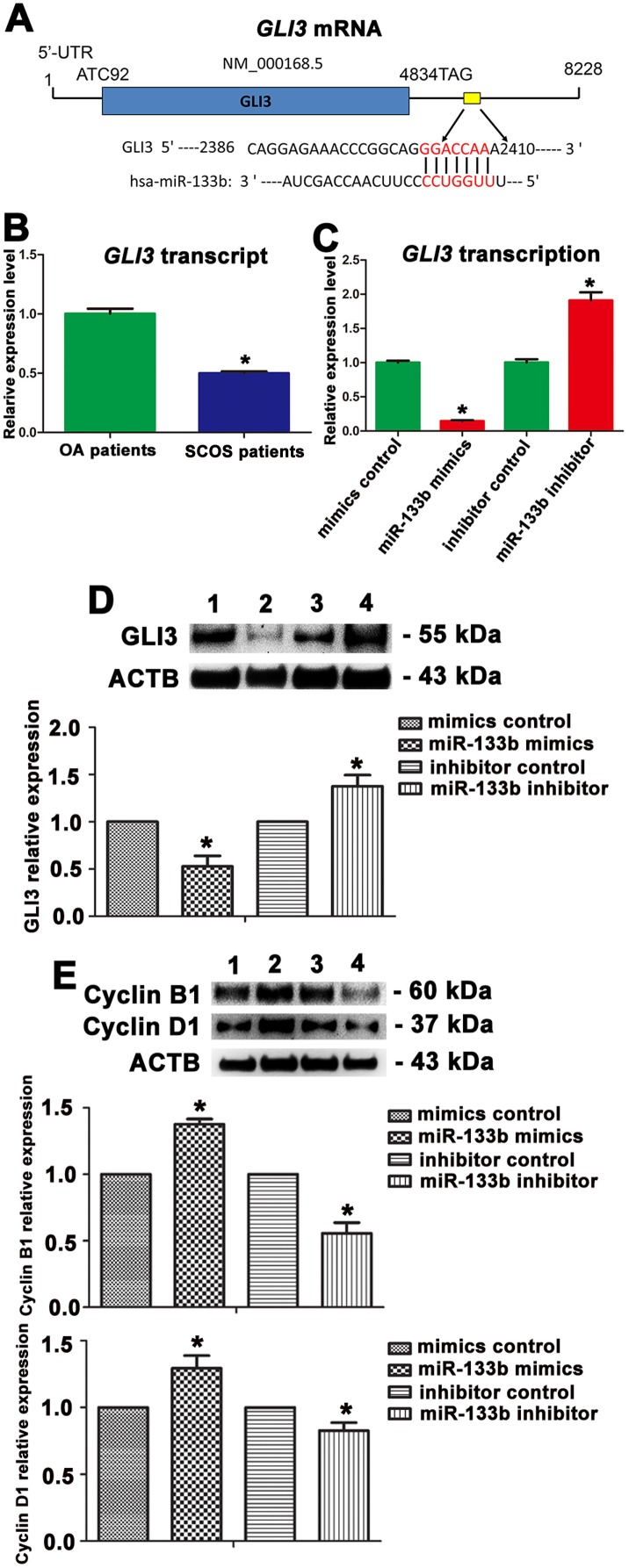
The expression changes of GLI3, Cyclin B1, and Cyclin D1 in human Sertoli cells treated with miR-133b mimic or inhibitor **A.** GLI3 is a predicted target and binding site of miR-133b in human Sertoli cells. **B.** Real-time PCR showed *GLI3* transcripts in human Sertoli cells between SCOS patients and OA patients. **C.** Real-time PCR revealed *GLI3* expression changes in human Sertoli cells at 48 hours after transfection of miRNA mimics control, miR-133b mimics, miRNA inhibitor control, and miR-133b inhibitor. **D.** Western blots demonstrated GLI3 expression in human Sertoli cells at 72 hours after transfection of miRNA mimics control (lane 1), miR-133b mimics (lane 2), miRNA inhibitor control (lane 3), and miR-133b inhibitor (lane 4) (upper panel). ACTB served as the control of loading proteins. The relative expression of GLI3 in human Sertoli cells at 72 hours after transfection of miR-133b mimics to miRNA mimics control and miR-133b inhibitor to miRNA inhibitor control after normalization to the signals of their loading control (low panel). **E.** Western blots revealed expression changes of Cyclin B1 and Cyclin D1 in human Sertoli cells at 72 hours after transfection of miRNA mimics control (lane 1), miR-133b mimics (lane 2), miRNA inhibitor control (lane 3), and miR-133b inhibitor (lane 4) (upper panel). ACTB was used as the control of loading proteins. The relative expression of Cyclin B1 and Cyclin D1 in human Sertoli cells at 72 hours after transfection of miR-133b mimics to miRNA mimics control and miR-133b inhibitor to miRNA inhibitor control after normalization to the signals of their loading control (middle and low panels). * indicated statistically significant differences (*p* < 0.05) between the miRNA-treated group and with the control.

To gain novel insights into molecular mechanisms underlying the function of miR-133b in regulating human Sertoli cells, we identified the targets of miR-133b. Using miRNA predict software, namely TargetScan, we predicted that transcription factor GLI3 was a binding target of miR-133b. As shown in Figure 7A, the 2^nd^-8^th^ nucleotides (the seed region) of miR-133b were base-pared with the 3′UTR sequence of GLI3. Real-time PCR showed that *GLI3* was expressed at a lower level in human Sertoli cell of SCOS patients than in these cells of OA patients (Figure 7B), which is contrast to the expression of miR-133b in human Sertoli cells of SCOS patients and OA patients. Furthermore, the transcripts of *GLI3* were remarkably decreased by miR-133b mimics (0.144±0.027) but significantly enhanced by miR-133b inhibitor (1.910±0.234) in human Sertoli cells (Figure 7C). Additionally, Western blots further revealed that GLI3 protein was obviously reduced by miR-133b mimics (0.528±0.194) in human Sertoli cells (Figure 7D); on the contrary, GLI3 translation was significantly elevated by miR-133b inhibitor (1.376±0.236) in human Sertoli cells (Figure 7D). Taken together, these data imply that GLI3 is a direct target of miR-133b in human Sertoli cells.

### MiR-133b stimulated the proliferation of human Sertoli cells via the activation of Cyclin B1 and Cyclin D1

Cell cycle proteins play important roles in regulating the entrance of cells to the S phase and cell proliferation [8]. We further examined whether miR-133b changed the expression of cell cycle regulators, including Cyclin B1 and Cyclin D1, in human Sertoli cells after transfection of miR-133b mimics and inhibitor. Western blots displayed that expression of Cyclin B1 (1.377±0.067) and Cyclin D1 (1.293±0.166) was elevated by miR-133b mimics, whereas the expression of these proteins was decreased by miR-133b inhibitor (Cyclin B1, 0.556±0.136; Cyclin D1, 0.827±0.100) (Figure 7E), indicating that Cyclin B1 and Cyclin D1 are activated by miR-133b in human Sertoli cells.

### GLI3 knockdown stimulated the growth of human Sertoli cells

**Figure 8:**
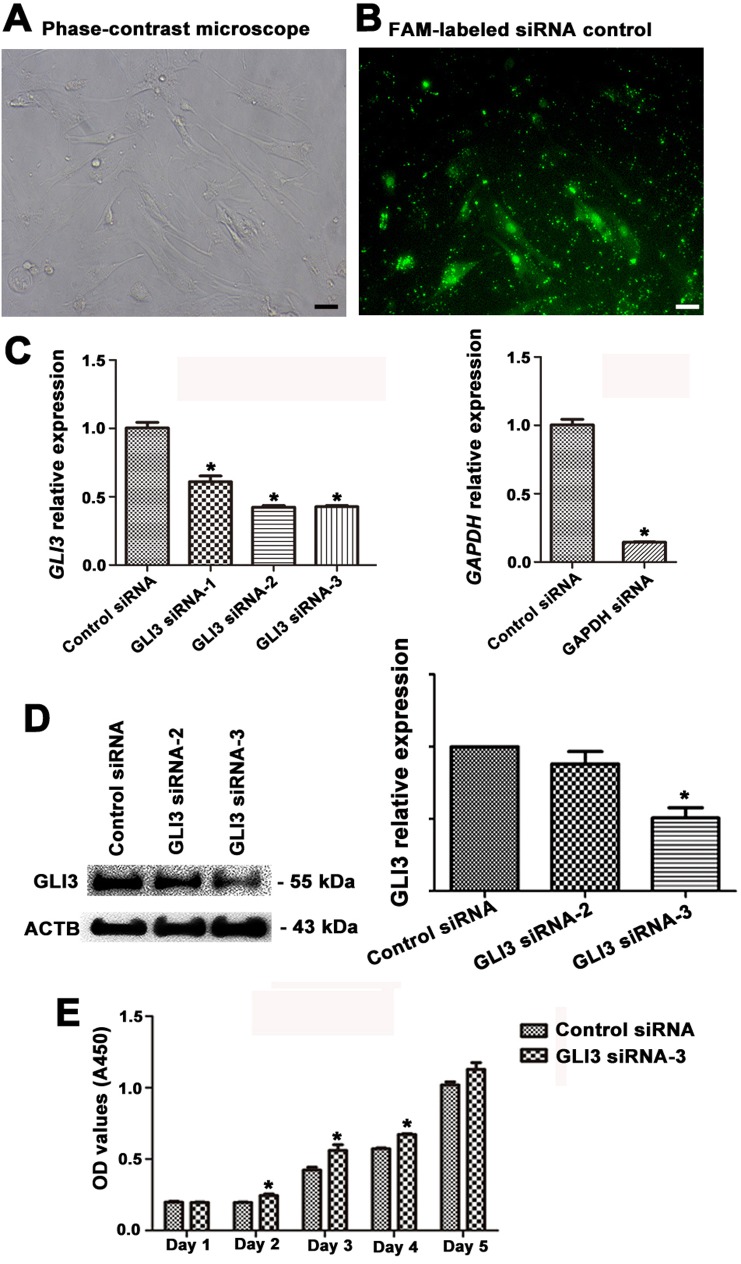
Transfection efficiency of GLI3 siRNAs and the influence of GLI3 knockdown on the growth of human Sertoli cells **A.**-**B.** Phase-contrast microscope **A.** and fluorescence microscope **B.** revealed transfection efficiency of GLI3 siRNAs using the FAM-labeled siRNA control. Scale bars in A-B = 20 μm. **C.** Real-time PCR showed the expression of *GLI3* (left panel) and *GAPDH* mRNA (right panel) in human Sertoli cells at 24 hours after transfection with control siRNA, GLI3 siRNA1-3, or GADPH siRNA, respectively. **D.** Western blots demonstrated the GLI3 protein expression in human Sertoli cells at 72 hours after transfection with control siRNA or with GLI3 siRNA-2 or GLI3 siRNA-3. ACTB was used as a loading control of proteins. **E.** CCK-8 assay showed the growth activity of human Sertoli cells after transfection of control siRNA or GLI3 siRNA-3 for 1 to 5 days. * indicated statistically significant differences (*p* < 0.05) between the siRNA-treated group and with the control.

We finally probed the role of miR-133b target GLI3 in human Sertoli cells. Gene silencing of GLI3 by RNA interference (RNAi) was performed to explore the function of GLI3 in regulating the growth of primary human Sertoli cells. The transfection efficiency of GLI3 siRNAs in Sertoli cells was over 80%, as evidenced by the transfection of FAM-labeled green fluorescent oligo (Figure 8A-8B). To obtain the sequence-specific siRNAs of GLI3, we assessed three pairs of GLI3 siRNAs (i.e., GLI3 siRNA-1, GLI3 siRNA-2, GLI3 siRNA-3) targeting different regions of GLI3 mRNA. Quantitative real-time PCR and Western blots revealed that siRNA-3 against GLI3 specifically knocked down the expression of *GLI3* mRNA (Figure 8C) and GLI3 protein (Figure 8D). Accordingly, we chose GLI3 siRNA-3 to further examine the effect of GLI3 on the proliferation of human Sertoli cells. Significantly, CCK-8 assays showed that GLI3 knockdown resulted in an obvious increase of human Sertoli cells via a time-dependent manner (Figure 8E), suggesting that GLI3 inhibits the propagation of human Sertoli cells.

## DISCUSSION

It is of great significance to examine the biology of Sertoli cells, which could help us gain novel insights into reproductive biology and cell-based therapy for treating human diseases. Human Sertoli cells can be cultured for a long term, whilst maintaining their primary morphology, stable global gene expression patterns, and a number of proteins [29], and GATA4-deficiency in Sertoli cells leads to progressive depletion of male germ cells [30]. We have revealed distinct characteristics of morphology and biochemical phenotype in human Sertoli cells between NOA patients and OA patients with normal spermatogenesis [13], which provides new information about genetic regulators causing non-obstructive azoospermia. Nevertheless, the differences in epigenetic regulators of human Sertoli cells between NOA and OA patients remain unknown. In this study, we found that 88 miRNAs were upregulated whereas 86 miRNAs were downregulated in human Sertoli cells of SCOS patients when compared to OA patients. Our real-time PCR data confirmed the quality and authenticity of our miRNA microarray data. These results could shed novel insights into epigenetic regulation underlying azoospermia including SCOS.

Growing evidence has indicated that miRNAs have critical function in regulating male germ cell development in rodents. It has been reported that miR-21 promotes the self-renew of Thy1^+^ mouse SSCs by the regulation of transcription factor EVT5 [31]. We have recently demonstrated that miR-20 and miR-106a play essential roles in regulating the proliferation and maintenance of mouse SSCs via targeting Stat3 [32]. Nevertheless, it is still unknown about the functions and targets of miRNAs in human Sertoli cells. In this study, we found, using miRNA microarray and real-time PCR, that miR-133b was upregulated in human Sertoli cells of SCOS patients compared to OA patients with normal spermatogenesis, suggesting that abnormal expression of miR-133b may be associated with pathogenesis of the SCOS.

In our view, miR-133b might be involved in the etiology of NOA including SCOS, since we found that miR-133b promoted the proliferation of human Sertoli cells. Our hypothesis can be supported by the findings that the proliferative activity of Sertoli cells is remarkably increased in cryptorchid testis compared to the control normal testis [33], suggesting that excessive propagation of human Sertoli cells may lead to a decreased number of male germ cells.

Nothing is known about the targets of miRNAs in human Sertoli cells. Using bioinformatics algorithms, we predicted that GLI3 was a binding target of miR-133b. GLI3 is an important transcription factor of Hedgehog (Hh) signal pathway that mediates cell proliferation and differentiation [34]. In rodents, Desert Hedgehog (Dhh) is highly expressed in Sertoli cells, and its expression is initiated at E11.5 in Sertoli cell precursors shortly after the activation of Sry and persists in the testis into adult Sertoli cells [35]. Adult male testes show a gross germ cell deficiency in *Dhh*-mutation mice [36]. The seminiferous tubules of most severe individuals are completely devoid of male germ cells, and only Sertoli cells retain, indicating that Dhh signal pathway may be associated with the etiology of SCOS. Hh signaling is mediated by Gli transcription factor in vertebrates, and Gli3 is proteolyzed to produce a repressor form to inhibit Hh expression [37]. The Dhh expression of adult Sertoli cells is very lower compared to embryonic stages [35]. Thus, Gli3 is presented as the repressor form in adult mouse Sertoli cells. In this study, we identified that GLI3 is indeed a direct target of miR-133b in human Sertoli cells. Functional assays revealed that gene silencing of GLI3 by RNAi stimulated the growth of human Sertoli cells, which was consistent with our data showing that miR-133b mimic promoted the proliferation of human Sertoli cells. To gain a deeper understanding of molecular mechanisms by which miR-133b regulates the fate determinations of human Sertoli cells, we identified two cell cycle regulators, including Cyclin B1 and Cyclin D1, as the indirect targets for miR-133b.

## CONCLUSIONS

In summary, we have for the first time compared global miRNA profiles in human Sertoli cells between SCOS patients and OA patients. We have identified that 174 miRNAs were differentially expressed in human Sertoli cells between SCOS patients and OA patients. These findings might provide novel epigenetic regulators for the pathogenesis of azoospermia and SCOS. We highlight that miR-133b, which was upregulated in Sertoli cells of SCOS patients, promoted the propagation of human Sertoli cells by targeting GLI3 and activating Cyclin B1 and Cyclin D1. Given Sertoli cells play key roles in regulating spermatogenesis and they have significant applications in regenerative medicine, this study could offer new insights into better understanding the underlying etiology for azoospermia and might offer new targets for developing novel approaches for treating male reproductive disorders and other human diseases including cancer. As example, miR-133b has been shown to stimulate the tumorgenesis and metastasis of human cervical carcinoma [23], and thus gene targeting for miR-133b and its target GLI3 might be used to treat cervical carcinoma and other tumors in the future.

## MATERIALS AND METHODS

### Procurement of testicular tissues from OA patients and SCOS patients

Testicular tissues were obtained from OA patients and SCOS patients who underwent microdissection testicular sperm extraction from February 2014 to November 2015 at Ren Ji Hospital affiliated to Shanghai Jiao Tong University School of Medicine. All OA patients were caused by the vasoligation or inflammation, and normal spermatogenesis was observed in these patients. The patients with SCOS were diagnosed by histological analysis showing that only Sertoli cells were present within the seminiferous tubules. This study was approved by the Institutional Ethical Review Committee of Ren Ji Hospital (license number of ethics statement: 2012-01), Shanghai Jiao Tong University School of Medicine, and the written informed consents for testicular biopsies were obtained from the donors for research only.

### Isolation and identification of human Sertoli cells from OA patients and SCOS patients

Testicular tissues from OA patients and SCOS patients were washed three times aseptically in Dulbecco modified Eagle medium (DMEM) (Gibco) containing antibiotic with penicillin and streptomycin (Gibco). Seminiferous tubules were separated from testis biopsies by the first enzymatic digestion consisting of 2 mg/ml collagenase IV (Gibco) and 1 μg/μl DNase I (Gibco) in 34°C water bath for 15 min. Sertoli cells and human male germ cells were obtained from seminiferous tubules using a second enzymatic digestion comprising 4 mg/ml collagenase IV, 2.5 mg/ml hyaluronidase (Sigma), 2 mg/ml trypsin (Sigma) and 1 μg/μl DNase I and followed by differential plating pursuant to the procedure as previously described [25]. For differential plating, cell suspension was seeded into matrigel^TM^ (BD Biosciences)-coated dishes in DMEM/F12 supplemented with 10% fetal bovine serum (FBS) (Gibco) and incubated at 34°C in 5% CO_2_ for 1 day. After incubation, the medium containing male germ cells were removed, and Sertoli cells attached to culture plates. The viability of freshly isolated human Sertoli cells was assessed by the exclusion of trypan blue staining. Freshly isolated human Sertoli cells were identified by RT-PCR and immunnostaining with antibodies against GATA4, WT1, SOX9, BMP4, SCF, and GDNF as described below.

### Immunocytochemistry

For immunocytochemical staining, human Sertoli cells were fixed with 4% paraformaldehyde (PFA) for 30 min, washed three times with cold phosphate-buffered saline (PBS) and permeabilized in 0.4% triton-X 100 (Sigma) for 5 min. After washing with PBS, the cells were blocked in 2% BSA for 30 min and followed by incubation with primary antibodies, including anti-GATA4 (Santa Cruz, catalog no: SC-1237, dilution: 1:200), anti-WT1 (Santa Cruz, catalog no: SC-192, dilution: 1:200), anti-SOX9 (Millipore, catalog no: AB5535, dilution: 1:100), anti-GDNF (Santa Cruz, catalog no: SC-328, dilution: 1:200), anti-SCF (Sigma, catalog no: SAB3500292, dilution: 1:200), anti-BMP4 (Abcam, catalog no: ab124715, dilution: 1:200), anti-CYP11A1 (Abcam, catalog no: ab75479, dilution: 1:200), and anti-smooth muscle actin (SMA, Abcam, catalog no: ab5694, dilution: 1:200 ) overnight at 4°C. Replacement of primary antibodies with isotype IgGs (Santa Cruz, at 1:50 dilutions) served as negative controls. After extensive washes with PBS for 30 min, the cells were incubated with the secondary antibody IgG (Sigma) conjugated with fluorescein isothiocyanate (FITC) or rhodamine at a 1:200 dilution for 1 hour at room temperature. DAPI (4, 6-diamidino-2-phenylindole) was used to label the nuclei, and images were captured with a fluorescence microscope (Nikon).

### Immunohistochemistry

Immunohistochemistry was performed according to the method as described previously [25]. In brief, testicular biopsies were obtained from OA and SCO patients. After being washed three times by PBS, the tissues were fixed with 4% PFA for 30 min. The biopsies were sectioned at 5 μm thickness. After washing with PBS, the sections were permeabilized in 0.5% triton-X 100 (Sigma) for 15 min and blocked in 5% BSA for 1 h and followed by incubation with primary antibodies including anti-SOX9 (Millipore catalog no: AB5535, dilution: 1:100) and anti-PCNA (Santa Cruz, catalog no: SC-7907, dilution: 1:200) overnight at 4°C. The slides were incubated with Alexa Fluor 555-labeled secondary antibody or Alexa Fluor 488-labeled secondary antibody at a 1:200 dilution for 1 h. DAPI was used to label the nuclei. Replacement of primary antibody with PBS was used as a negative control.

### RNA extraction and reverse transcription-polymerase chain reaction (RT-PCR)

Total RNA was extracted from human Sertoli cells using the Trizol reagent (Invitrogen), and the quality and concentrations of total RNA were measured by Nanodrop. Reverse transcription (RT) was performed using the First Strand cDNA Synthesis Kit (Thermo Scientific, catalog no: K1622), and PCR of the cDNA was carried out according to the protocol as described previously [38]. The primers of the chosen genes, including *GATA4* (GATA binding protein 4), *WT1* (Wilms tumor 1), *SOX9* (Sex Determining Region Y-Box 9), *GDNF*, *BMP4*, *SCF*, *FSHR* (follicle-stimulating hormone receptor), *AR* (androgen receptor), and *ACTB* (actin beta), were designed and listed in Table 1. The PCR reaction started at 94°C for 2 min and was performed as follows: denaturation at 94°C for 30 sec, annealing at 55-60°C for 45 sec as listed in Table 1, and elongation at 72°C for 45 sec. After 35 cycles, the samples were incubated for an additional 5 min at 72°C. PCR products were separated by electrophoresis on 2% agarose gel and visualized with ethidium bromide. Images were recorded and band intensities were analyzed using chemiluminescence (Chemi-Doc XRS, Bio-Rad). RNA without RT (RT-) but with PCR of *ACTB* primers served as a negative control.

### Quantitative real-time PCR

RNA was extracted from human Sertoli cells of patients with OA and SCOS patients, or Sertoli cells with miR-133b mimics, miR-133b inhibitor, miRNA mimics control, miRNA inhibitor control, using Trizol reagent (Invitrogen). For miRNA real-time PCR, RT reaction was performed using miScript® II RT Kit (Qiagen, catalog no: 218160). Each RT reaction was composed of 100 ng RNA, 4 μl of miScript HiSpec Buffer, 2 μl of Nucleics Mix, and 2 μl of miScript Reverse Transcriptase Mix (Qiagen), in a total volume of 20 μl. Reactions were performed in a Veriti^®^ 96-Well Thermal Cycler (Applied Biosystems) for 60 min at 37°C, and followed by heat inactivation of RT for 5 min at 95°C. RT reaction mix was diluted by 5 times in nuclease-free water and held at −20°C. Primer sequences of miRNAs used for real-time PCR were listed in Table 2. Real-time PCR was performed in triplicate using 7500 Fast Real-Time PCR System (Applied Biosystems) with 25 μl of PCR reaction mixture containing 2 μl of cDNA, 12.5 μl of QuantiTect SYBR Green PCR Master Mix (Qiagen), 2.5 μl of universal primer (Qiagen), 2.5 μl of miRNA-specific primer (Table 2), and 5.5 μl of nuclease-free water. Reactions were incubated in a 96-well optical plate (Applied Biosystems) at 95°C for 10 min, and followed by 40 cycles of 95°C for 10 sec, 60°C for 30 sec. Individual samples were run in triplicate. In the end of the PCR cycles, melting curve analysis was performed to validate the specific generation of the expected PCR products. The expression levels of miRNAs were normalized to U6 and calculated using the 2^−ΔΔCt^ method [38].

Real-time PCR was also carried out to evaluate the expression of *GLI3* (GLI family zinc Finger 3) in freshly isolated human Sertoli cells from OA patients and SCOS patients or the expression of *GLI3* and *GAPDH* in human Sertoli cells after transfection of GLI3 siRNAs or GAPDH siRNA pursue to the method described previously [14]. The primers of these genes were designed and listed in Table 1, and their expression levels were normalized to *ACTB* and calculated using the 2^−ΔΔCt^ method.

### MiRNA microarrays

Total RNA was extracted from the freshly separated human Sertoli cells of OA patients and SCOS patients using the mirVanaTM RNA Isolation Kit (Ambion, catalog no: AM1561). The quality of total RNA was checked by gel imaging and electropherogram, and RNA integrity number (RIN) was used to assess RNA quality showing RIN values equaling or over 7.0. MiRNA microarrays were conducted on Agilent Human miRNA V21.0 (Oebiotech, Shanghai, China), and representative miRNA microarray data confirmed by real-time PCR were shown. The sample labeling and microarray hybridization were performed according to the manufacturer's manual. Briefly, total RNA were dephosphorylated, denaturated, and labeled with Cyanine-3-CTP. The labeled RNAs were hybridized onto the miRNA arrays. After extensive washes, the arrays were scanned with the Agilent Scanner G2505C (Agilent Technologies). The significantly differentially expressed miRNAs were selected according to the criteria:log_2_(Fold change) ≧0.585.

### Transfection of miRNA mimics, miRNA inhibitors, GLI3 siRNAs and GAPDH siRNA into human Sertoli cells

The miRNA mimics and inhibitors were purchased from GenePharma (Shanghai, China). The oligonucleotides of miRNA mimics and inhibitors were listed in Table 3. Human Sertoli cells were seeded at 2×10^5^/cm^2^ density and cultured in DMEM/F12 supplemented with 10% FBS for overnight. The medium was changed to DMEM/F12 supplemented with 1% FBS.

Sertoli cells were classified into four groups in term of transfecting different miRNAs: i) miRNA mimics control, ii) miR-133b mimics, iii) miRNA inhibitor control, and iv) miR-133b inhibitor. For RNA interference assays, Sertoli cells were classified into five groups based on transfecting different siRNAs: i) control siRNA, ii) GLI3 siRNA-1, iii) GLI3 siRNA-2, iv) GLI3 siRNA-3, and v) GAPDH siRNA. Transfection of miRNA mimics or inhibitor and siRNAs were conducted using lipofectamine 2000 transfection agent (Invitrogen) according to the method as described previously [14]. Different concentrations of miR-133b mimics, inhibitor and GLI3 siRNAs were utilized to optimize the transfection efficiency, and we found that 40 μM of miR-133b mimics, inhibitor and GLI3 siRNAs were sufficient for their long-term biological effect. After 48 hours and 72 hours of culture, cells were harvested for examining the expression changes of various genes and proteins accordingly.

### Cell proliferation assays

Human Sertoli cells and a human SSC line [28] were seeded at a density of 1,000 cells/well in 96-well microtiter plates in DMEM/F12 supplemented with 1% FBS, and they were transfected with miRNA mimics control, miR-133b mimics, miRNA inhibitor control, miR-133b inhibitor, or GLI3 siRNA-3 or control siRNA. After 5 days of culture, the proliferation potential of human Sertoli cells was detected by CCK-8 assay (Dojin Laboratories, catalog no: CK04) according to the manufacturer's instruction.

### Western blots

Human Sertoli cells with miR-133b mimics or inhibitor treatment were lysed with RIPA buffer (Santa Cruz) for 30 min on ice. After 30 min of lysis, cell lysates were cleared by centrifugation at 12,000 g for 20 min, and the concentrations of proteins were measured by BCA kit (Dingguo Company, catalog no: P0012). Thirty micrograms of cell lysate from each sample were used for SDS-PAGE (Bio-Rad Laboratories), and Western blots were performed according to the protocol as described previously [8]. The chosen antibodies included anti-GLI3 (Sigma, catalog no: WH0002737M1, dilution: 1:500), anti-Cyclin B1 (Santa Cruz, catalog no: SC-752, dilution: 1:200), anti-Cyclin D1 (Santa Cruz, catalog no: SC-717, dilution: 1:200), anti-PCNA (Santa Cruz, catalog no: SC-7907, dilution: 1:200), and anti-ACTB (Protein tech, catalog no: HRP-60008, dilution: 1:5000). After extensive washes with TBST, the blots were detected by chemiluminescence (Chemi-Doc XRS, Bio-Rad).

### Statistical snalysis

All data were presented as mean ± SEM from at least three independent experiments and analyzed by Student's t-test or one-way ANOVA with the appropriate post-hoc tests (Dunnet's test or Turkey's multiple comparison) using Prism (version 5, GraphPad Software), and p< 0.05 was considered statistically significant.

## SUPPLEMENTARY FIGURES AND TABLES


