# Supplementary material for: MiRNA-133b promotes the proliferation of human Sertoli cells through targeting GLI3
Source: Oncotarget. 2016 Jan 10;7(3):2201–19. doi: 10.18632/oncotarget.6876 (PMC4823029; doi:10.18632/oncotarget.6876)
Supplement: Supplementary file 1 [file oncotarget-07-2201-s001.pdf]

MiRNA-133b promotes the proliferation of human Sertoli cells through targeting GLI3

Supplementary Material

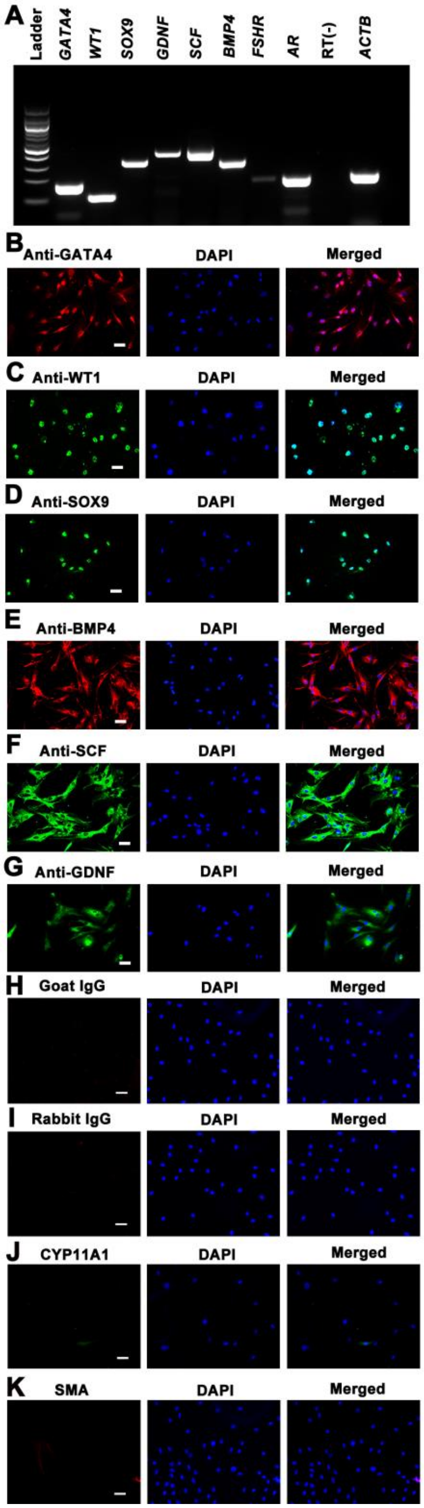

Supplementary Figure 1. Identification and characterization of freshly isolated human Sertoli cells. (**A**) RT-PCR showed the expression of numerous genes, including *GATA4*, *WT1*, *SOX9*, *GDNF*, *SCF*, *BMP4*, *FSHR* and *AR,* in the freshly human Sertoli cells. *ACTB* was used as a loading control of total RNA, whereas RNA samples without RT(-) but with PCR using *ACTB* primers served as a negative control. (**B**-**I**) Immunocytochemistry revealed the expression of GATA4 (**B**), WT1 (**C**), SOX9 (**D**), BMP4 (**E**), SCF (**F**), GDNF (**G**), goat IgG (**H**), rabbit IgG (**I**), CYP11A1 (**J**), and SMA (**K**) in the isolated human Sertoli cells. Scale bars in B-K = 20 μm.

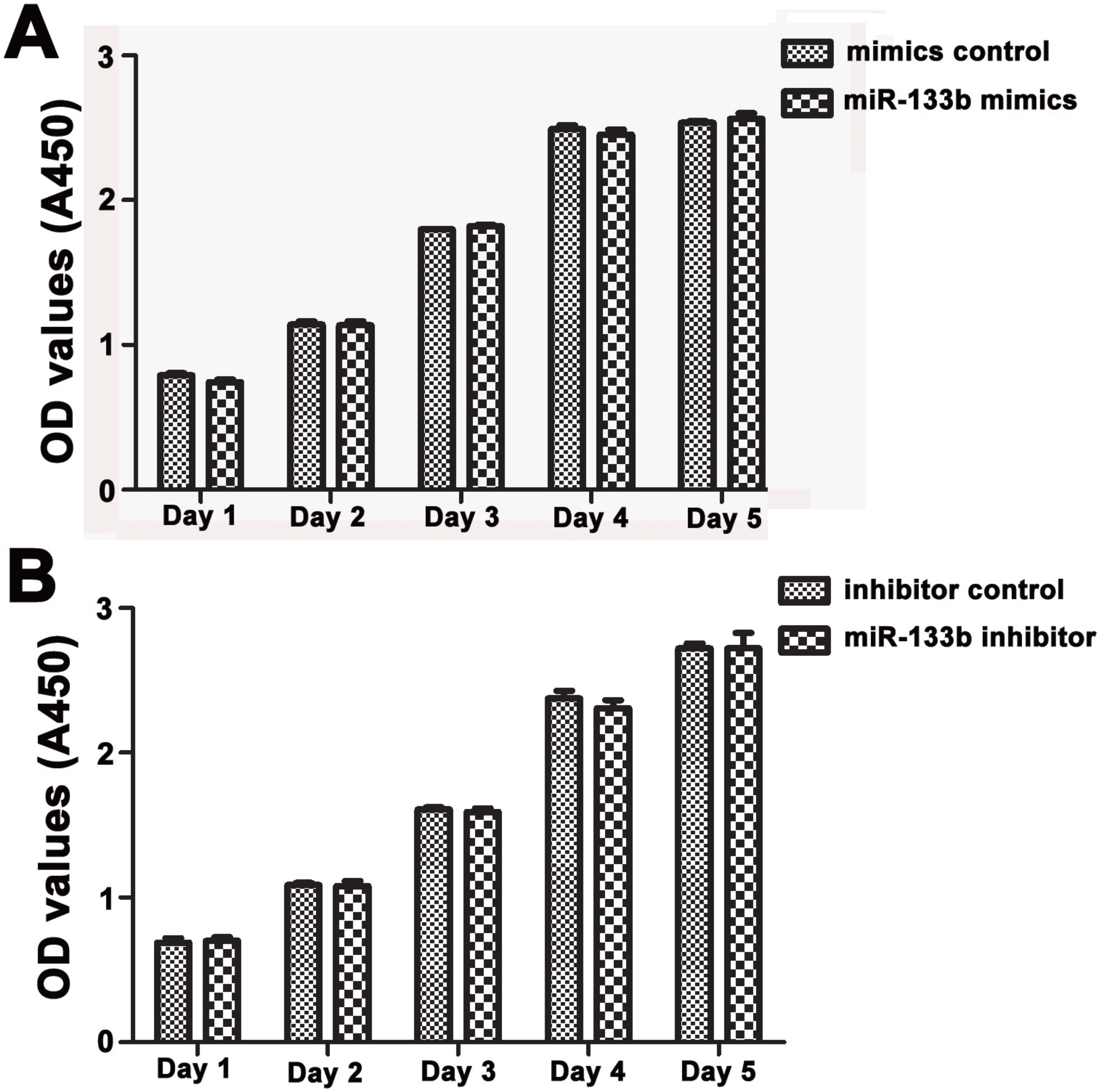

Supplementary Figure 2. The effect of miR-133b on the proliferation of human SSC line. (**A**-**B**) CCK-8 assay showed the growth curve of human SSC line treated with miRNA mimics control or miR-133b mimics for 5 days (**A**), and miRNA inhibitor control or miR-133b inhibitor for 5 days (**B**).
